# Contradiction or harmony? Spatial and temporal relationships between new urbanization and rural revitalization in the Yellow River Basin from a coupling perspective

**DOI:** 10.1371/journal.pone.0288600

**Published:** 2023-07-20

**Authors:** Guorong Qiao, Li Wang, Peng Du

**Affiliations:** School of Geography, Liaoning Normal University, Dalian, China; Qufu Normal University, CHINA

## Abstract

Integrating urban and rural areas is the only way to achieve sustainable regional development. To comprehensively build an evaluation index system for new urbanization and rural revitalization (NU-RR), taking the Yellow River Basin (YRB) as research object, and the coupled coordination degree (CCD) model, relative development degree (RDD) model and gravity model are used to quantitatively measure the spatial and temporal patterns, synchronous development status and spatial linkages of the coupled coordination of NU-RR from 2005 to 2020. The factors influencing the CCD are identified with the help of the geographic detector model. The findings indicate that: (1) From 2005 to 2020, the combined indexes of NU-RR in the YRB show an increasing trend, while rural revitalization is growing slower than new urbanization. (2) The CCD of NU-RR in the YRB shows spatial structure characterized by “high in the east and low in the west” and undergoes an evolutionary process of “low coupling-medium coupling-high coupling”. (3) The spatial disparities in the state of coupled and coordinated development of different cities are significant, mainly showing the spatial distribution characteristics dominated by the lagging new urbanization. (4) The spatial connection of CCD is networked and polarized, and the interprovincial barrier effect is weakened. (5) Total retail sales of consumer goods per capita and local general public budget expenditure as a share of GDP are the primary influencing elements affecting the CCD of NU-RR in the YRB. The interaction is manifested as bivariate enhance and nonlinear enhancement. The study’s findings can guide decisions to promote high-quality urban-rural integration development in the YRB.

## 1. Introduction

Urbanization is a vital force in the modernization of countries and regions. According to the UN Sustainable Development Agenda 2030, more than 50% of the global population resides in cities, and the world urbanization rate is expected to approach 60% by around 2030 [1]. Rapid urbanization, which has been accompanied by rapid economic growth, has not only put enormous strain on the environment and its resources, leading to several ecological issues [2], such as traffic congestion, water shortage [3], air pollution [4, 5] and declining ecosystem services [6]. It also severely hinders the sustainable growth between city and country areas while creating a vast divide in infrastructure, human living conditions, and public service facilities [7]. By the end of 2021, urbanization in China increased from 17.92% at the start of the reform and opening to 64.72%, generating a miracle of rapid global urbanization [8]. However, with the decline of the “village in the city”, rural hollowing and increasingly severe “rural disease” and other disorders are becoming more and more prominent in China, which poses an obstacle to the sustainable development of China [9]. In this situation, it is explicitly suggested to “implement the strategy of rural revitalization” in the report of the 19th Party Congress. It is further emphasized in the report of the 20th Party Congress to adhere to the coordinated development of urban and rural areas, smooth the flow of urban and rural elements, and promote the revitalization of rural development. These reports have indicated the direction for reshaping the new relationship between urban and rural workers and farmers under the new situation.

NU-RR are a symbiotic and co-prosperous community of destiny, and the deep integration of the two is the essential focal point for building a new type of industrial-agricultural relationship. New urbanization is the power source of rural revitalization, while rural revitalization is the endogenous power of new urbanization. New urbanization guides the transfer of urban talent, capital, technology and other factors to rural areas, stimulates rural economic potential, encourages the modernization and restructuring of rural enterprises, and realizes the optimal allocation of rural resource factors, etc. Rural revitalization provides a spatial carrier for the construction of new urbanization, expands the scale of towns, and provides cities with agricultural products, labor, raw materials and other elements. The development of cities will inevitably have a radiating effect on the countryside, and the development of the countryside can also force the cities to accelerate their development. Thus, studying the relationship between NU-RR is academically significant and practically significant.

Multiple aspects of the relationship between NU-RR, including one-way influence and mutual connection, have been explored in previous studies. Regarding one-way influence, both domestic and international researchers have focused on the issue of urbanization on rural development, mainly focusing on the impact effects of urbanization on rural revitalization [10], including positive effects [11] and adverse effects [12], rural settlement distribution characteristic [13], rural tourism [14], rural environmental perception [15], and so on. The two-way relationship between NU-RR is mainly reflected in urban-rural coupling and coordination. Most scholars believe that there is a coupling relationship between the two that mutually promotes and complements each other [16]. Xu et al. measured the CCD of NU-RR in China using the CCD model. They found that the CCD of most provinces and cities was at the stage of primary coordination and above, and the number of advanced coordination areas continued to increase [17]. Zhang et al. analyzed the interactive relationship between NU-RR using the mean squared difference decision method, CCD model and trend surface analysis [18]. Using Zhejiang Province as a research case, Yu et al. concluded that for every 1% increase in the rural revitalization index, the new urbanization index would increase by 0. 9414%, and the two have lasting synergy [19]. Similar conclusions were reached by Sun et al. [20]. There is also a series of studies on urban-rural relationship theory [21] and development model [22], which further enrich the research content of NU-RR.

In addition to coupling coordination evaluation of the two major systems, scholars have analyzed the primary influencing factors of CCD using various methods such as decoupling and traditional econometric regression models. Decoupling models provide the possibility to investigate the role of subsystems in coupling coordination or barrier factors from an internal perspective [23], mainly incorporating the Dagum Gini coefficient decomposition [24] and the Tapio decoupling model [25]. Traditional econometric regression methods include geographically weighted regression models [26], GMM estimation methods [27], fixed effects models [28], and spatial econometric models [29]. The study on the influencing factors discovered that many factors, including economic development, government service capacity, industrial structure, convenient transportation, consumption level, human capital, and agricultural production efficiency, influence the CCD between NU-RR.

As an important strategic region of the country, the YRB has an important position in the national economic development pattern. Existing studies mainly focus on the coupling relationship between new urbanization and the ecological environment [30], green development efficiency [31] and science and technology innovation [32], etc. There is a lack of research on the coupling relationship between urban and rural areas. The State Council published the Outline of Ecological Preservation and High-Quality Development of the YRB in October 2021, which emphasizes that the quality of the YRB is the most significant weakness and needs to be improved. Adhering to the coordinated growth of diverse regions, locations, and groups is essential to achieving high-quality development. As a result, improving the current state of people’s livelihood development in the YRB and fostering the all-encompassing and coordinated development of urban and rural areas is a serious problem that has to be resolved in the YRB.

Overall, the research on the relationship between NU-RR has yielded rich results. However, there is still a need for study in terms of research breadth, scope, and methodology from the coupling perspective. First, regarding the breadth of the research, the current studies only pay attention to the static CCD of a region, ignoring the crucial aspect of the spatial correlation of CCD between urban and rural areas in various regions. Secondly, in terms of research scale, most studies study the coupling relationship between NU-RR based on the national or provincial scale. Still, little attention has been paid to the watershed scale, especially the YRB, an important national strategic area. Thirdly, in terms of research methods, existing studies focus on a single influencing factor for the coupling and coordination of NU-RR, and lack research on the interaction of factors leading to spatial differentiation. Therefore, this study evaluates the coupling and coordination of NU-RR based on the data of 99 prefecture-level cities in the YRB from 2005 to 2020 using the CCD model, RDD model and gravity model. On this basis, the influence factors of the CCD are analyzed using the geographic detector model.

This article is structured as follows. Section 2 describes the study design and study area. Research methods in Section 3. Results analysis for Section 4. Section 5 analysis of influencing factors. Section 6 provides a discussion of the article and draws the main conclusions.

## 2. Study design and area

### 2.1 Study design

**Fig 1:**
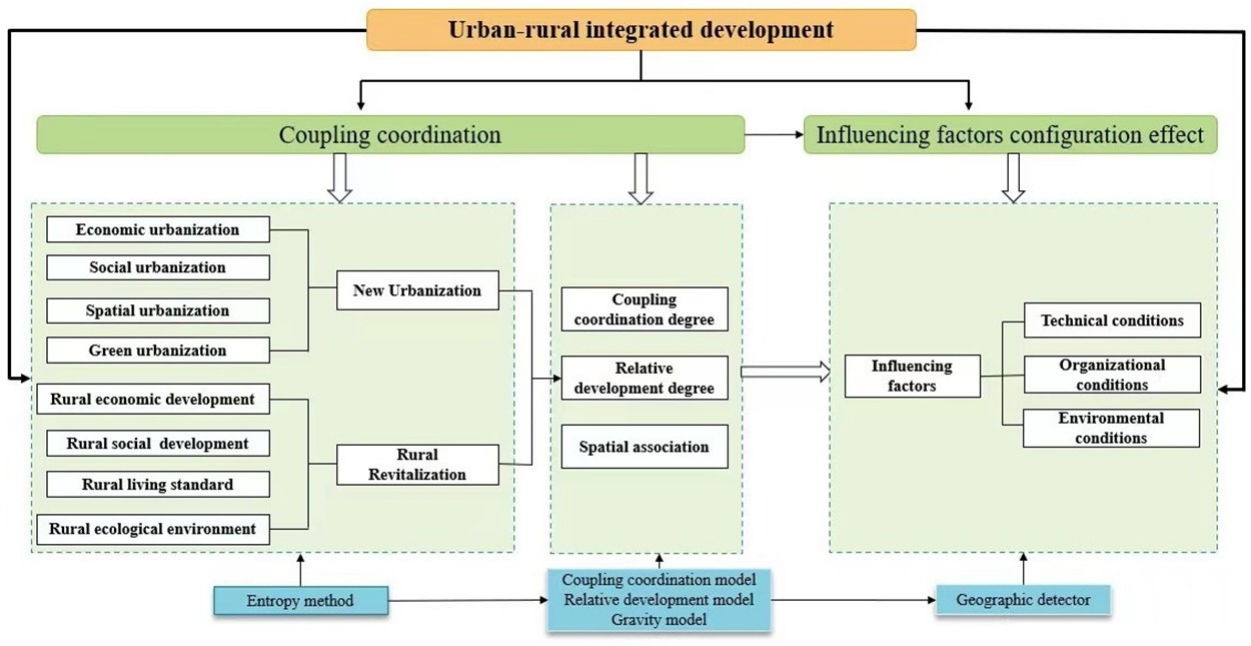
Study design.

This paper’s study design and the application approach are depicted in Fig 1, which mainly includes two sections: (1) Coupling coordination analysis. Firstly, to build an evaluation index system for NU-RR. Secondly, the entropy method calculates the index weights of NU-RR. Lastly, the coupling and coordination level, relative development type, and spatial connection intensity of NU-RR in the YRB are analyzed using the CCD, RDD, and gravity models. (2) Influencing factors analysis. Based on the TOE theoretical framework, the factors affecting the coupling and coordination of the two are selected and then profiled using a geographic detector.

### 2.2 Study area and data sources

**Fig 2:**
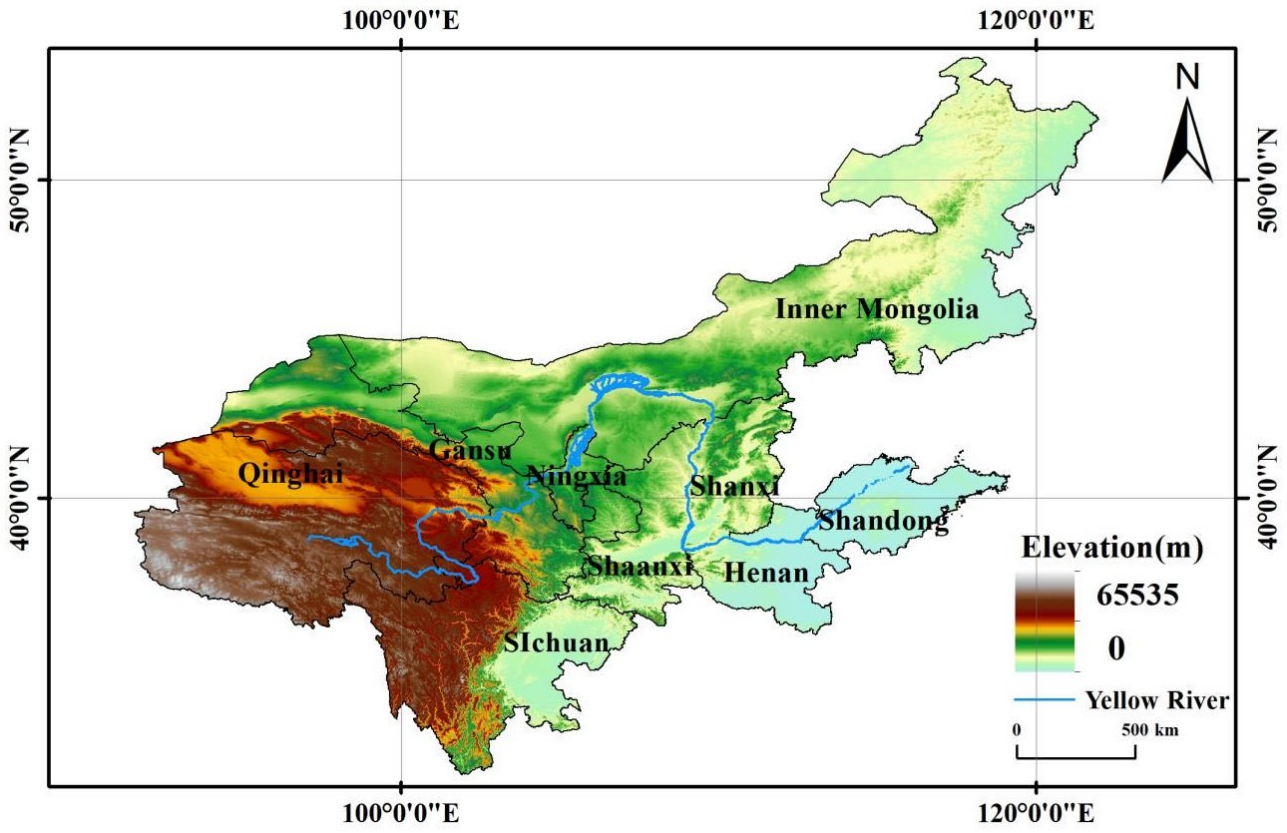
The study area (YRB). Shanxi Province (Taiyuan, Datong, Yangquan, Changzhi, Jincheng, Shuozhou, Jinzhong, Yuncheng, Xinzhou, Linfen, Luliang); Inner Mongolia (Hohhot, Baotou, Wuhai, Chifeng, Tongliao, Erdos, HulunBeier, Bayannur, Ulanqab); Shandong Province (Jinan, Qingdao, Zibo, Zaozhuang, Dongying, Yantai, Weifang, Jining, Tai’an, Weihai, Rizhao, Linyi, Dezhou, Liaocheng, Binzhou, Heze); Henan Province (Zhengzhou, Kaifeng, Luoyang, Pingdingshan, Anyang, Hebi, Xinxiang, Jiaozuo, Puyang, Xuchang, Luohe, Sanmenxia, Nanyang, Shangqiu, Xinyang, Zhoukou, Zhumadian); Sichuan Province (Chengdu, Zigong, Panzhihua, Luzhou, Deyang, Mianyang, Guangyuan, Suining, Neijiang, Leshan, Nanchong, Meishan, Yibin, Guang’an, Dazhou, Ya’an, Bazhong, Ziyang); Shaanxi Province (Xi’an, Tongchuan, Baoji, Xianyang, Weinan, Yan’an, Hanzhong, Yulin, Ankang, Shangluo); Gansu Province (Lanzhou, Jiayuguan, Jinchang, Baiyin, Tianshui, Wuwei, Zhangye, Pingliang, Jiuquan, Qingyang, Dingxi, Longnan); Qinghai Province (Xining); Ningxi (YingChuan, Shizuishan, Wuzhong, Guyuan, Zhongwei).

YRB refers to the entire Yellow River system, from its source to the ocean. This river has an impact on the area’s geography and ecology since it crosses the three significant geographic gradients of east, central, and west while flowing through the provinces of Qinghai, Gansu, Ningxia, Inner Mongolia, Shaanxi, Shanxi, Henan, Shandong, and Sichuan (regions) (Fig 2). At the end of 2019, the YRB had a total population of 442 million, or 31.58% of the country, a watershed area of nearly 750,000 square kilometers, a land area of 37% of the nation, and a regional GDP of 24.74 trillion yuan, or roughly 25% of the nation. Yet the GDP per capita is only 60,000 yuan, lower than the 70,000 yuan national average, with significant differences between different cities [23]. The YRB is an area in China with a comparatively concentrated poor population and extremely imbalanced urban and rural growth due to its fragile ecosystem background and relatively sluggish social and economic growth. Therefore, we must be given full attention.

The information required for this study’s calculations was acquired from the 2006–2021 China Urban Statistical Yearbook, the China Urban and Rural Construction Statistical Yearbook, and the yearbooks for nine provinces (cities and autonomous regions) in the YRB. The PM2.5 pollution data is derived from the global PM2.5 satellite raster data published by Columbia University’s Center for Socioeconomic Data and Applications. Leagues, autonomous areas, and cities with more missing data were ultimately excluded, leaving 99 prefecture-level cities in the YRB to be included in the study, and some cities with missing data were filled in using interpolation of adjacent years. Base map from National Center for Basic Geographic Information (http://www.ngcc.cn/ngcc/).

## 3. Research methods

### 3.1 Constructing indicator system

#### 3.1.1 Indicators are chosen for the NU-RR

Relevant studies on new urbanization have been abundant. Referring to related research results [33, 34] and following the principle of scientific applicability, a new urbanization index system is created from four dimensions: economic, social, spatial, and green, as shown in Table 1. Using per capita GDP, the proportion of output value of the tertiary industry, and per capita disposable income of urban residents to indicate economic urbanization. Social urbanization primarily refers to the extent to which public services like healthcare, education, and infrastructure have improved and is characterized by education expenditure and the medical personnel per 10,000 people. In spatial urbanization, the area of built-up areas in towns is selected to measure the size of cities, and the congestion of urban roads is measured using the area of urban roads per capita. Green urbanization is indicated using the green coverage rate of built-up areas, the centralized treatment rate of sewage treatment plants, and the area of green park space. The construction of indicators for rural revitalization has yet to form a unified standard. This paper refers to the existing research [35–37] and constructs an evaluation indicator system for rural revitalization from four dimensions: economic development, social development, living standard, and ecological environment, as shown in Table 1. Rural economic development is characterized by the share of agricultural, forestry, livestock and fishery output and food production, which visually reflect the level of agricultural development. The total power of agricultural machinery and rural employment is used to measure the degree of social development of villages. The income and expenditure of rural residents express the rural living standard. Fertilizer application and PM2.5 air quality reflect the pollution level of the rural soil and air environments and are used to characterize the rural ecological environment. This work standardizes the original data and uses the entropy value approach to find the index weights to remove the influence of dimensionality and dimensionality. The precise calculation method is presented in Eqs (1–3).

**Table 1:** The evaluation indicator system.

System	Subsystems	Indicator	Weight	Type
New urbanization	Economic urbanization	Per capita GDP /yuan	0.1244	+
		Proportion of output value of tertiary industry /%	0.0357	+
Rural revitalization		Per capita disposable income of urban residents /yuan	0.0918	+
	Social urbanization	Education expenditure /yuan	0.1554	+
		Medical personnel per 10,000 people / people	0.0879	+
	Spatial urbanization	Urban built-up area /km^2^	0.1908	+
		Per capita urban road area /m^2^	0.0607	+
	Green urbanization	Green coverage rate of built-up area /%	0.0152	+
		Centralized treatment rate of sewage treatment plant /%	0.0246	+
		Park green space area / hectare	0.2136	+
	Rural economic development	Agriculture, forestry, animal husbandry and fishery output value proportion /%	0.1195	+
		Grain yield /Tons	0.1839	+
	Rural social development	Agricultural machinery total power / Million kilowatts	0.1952	+
		Rural employment personnel / Ten thousand people	0.1714	+
		Per capita living consumption expenditure of rural residents /yuan	0.1376	+
	Rural living standard	Per capita net income of rural residents /yuan	0.1210	+
		Fertilizer application rate /Tons PM_2.5_ air quality /um	0.0219	-
	Rural ecological environment	Per capita disposable income of urban residents /yuan	0.0495	-

#### 3.1.2 Factors influencing the CCD of NU-RR

Based on the existing research results [33, 38], the “Technology-Organization-Environment” (TOE) framework, which is widely used to find the influencing factors of public policy, is used to divide the influencing factors of the CCD of NU-RR into three parts (Table 2).

**Table 2:** Index system of influencing factors.

Dimension	Influencing factor	Indicator	Description
Technical conditions	Economic Development	GDP per capita (*X*_*1*_)	GDP/year-end total population (yuan/person)
		Rural-urban income ratio (*X*_*2*_)	Urban disposable income/rural per capita net income(%)
	Industrial Structure	Secondary industry as percentage to GDP(*X*_*3*_)	Output value of secondary industry /GDP (%)
		Tertiary industry as percentage to GDP(*X*_*4*_)	Output value of tertiary industry /GDP (%)
Organizational conditions	Government Support	Local general public budget expenditures as percentage to GDP(*X*_*5*_)	Expenditures in local general public budgets /GDP (%)
		Expenditure on science and technology as percentage of GDP(*X*_*6*_)	Science and technology expenditure/GDP(%)
	Human Resource	Population density (*X*_*7*_)	Year-end total population /administrative area (person/km^2^)
		Number of students enrolled in general higher education schools by 10,000 people(*X*_*8*_)	Number of students enrolled in general higher education schools/ year-end total population (number/ person)
Environmental conditions	Social Environment	Fixed asset investment per capita(*X*_*9*_)	Fixed asset investment/year-end total population(yuan/person)
	Market environment	Total retail sales of social consumer goods per capita(*X*_*10*_)	Total retail sales of consumer goods/year-end total population(yuan/person)
	Information level	Number of international Internet users by 10,000 people(*X*_*11*_)	Number of international Internet users/ year-end total population (number/ person)

Technical conditions primarily represent technological refers to the technology available to enable urban-rural development, including economic development and industrial structure [39]. Economic growth provides financial and technological support for urban-rural integration, enabling the rapidly developing side of the economy to play a spatial spillover role. In addition to facilitating the transformation and upgrading of physical industries in towns and cities, optimizing and upgrading industrial structures, particularly the development of secondary and tertiary sectors, aids agriculture in realizing large-scale and modernized operations. It allows the rationalization and advanced industrial structure to be continuously improved.

Organizational conditions mainly involve organizational support capabilities (structure, size, and institutions) and focus on fundamental needs in urban-rural areas, including government support and human resources [40]. In general, investments in city and countryside public infrastructure, particularly in rural buildings, increase with local general public budget expenditures. Government support is crucial in enhancing rural infrastructure and alleviating poverty, as rural development is susceptible to a cycle of poverty. Human capital is required to facilitate the high-quality integrated development of urban and rural communities. More educated people can better master the significance of urban and rural co-development, learn fundamental scientific concepts, and motivate farmers to explore positive development models to narrow the urban-rural disparities.

Environmental conditions mostly pertain to the outside environment, including society, the market, and the level of information. The social environment refers to the fixed asset investment condition. Fixed asset investments are essential to socialized reproduction, strengthening the economy and bridging the earnings gap between urban-rural dwellers. The vigorous and sustainable development of the market can promote the rational allocation of urban-rural resources and bring new impetus to urban-rural development. The development of urban and rural areas together is given new life by the degree of information technology. It aids in removing the information “barrier” that separates urban and rural communities.

### 3.2 Research methods

#### 3.2.1 Composite index calculation

Each index is normalized using the maximum-minimum standardization method. The weights used to generate the comprehensive indices of NU-RR are then determined using the entropy weight method. The following are the formulas:

$$\text{Positive}\;\text{indicators}:X_{\text{ij}}=\frac{x_{ij}-\text{min}\;x_{ij}}{\text{max}\;x_{ij}-\text{min}\;x_{ij}}+0.000001$$ (1)

$$\text{Negative}\;\text{indicators}:y_{\text{ij}}=\frac{\text{max}\;x_{ij}-y_{ij}}{\text{max}\;y_{ij}-\text{min}\;y_{ij}}+0.000001$$ (2)

$$W_{j}=\frac{1-E_{j}}{\sum_{i=1}^{m}\left(1-E_{j}\right)}$$ (3)

$$X_{i}=\sum_{j=1}^{m}w_{ij}X_{ij}$$ (4)

$$Y_{i}=\sum_{j=1}^{m}w_{ij}Y_{ij}$$ (5)

Where: *X*_*ij*_, *y*_*ij*_ are the standardized data, denoting the standardized values of the ith sample and the jth index (i = 1, 2, 3, …, n; j = 1, 2, 3, …, m), max *x*_*ij*_ and min *x*_*ij*_ are highest and lowest values of the corresponding indicator data. *W*_*j*_ denotes the weights of each variable; *X*_*i*_ represents the composite index of new urbanization; *Y*_*i*_ denotes the composite index of rural revitalization.

#### 3.2.2 CCD model

Coupling coordination describes the level of mutual coordination among two systems throughout development and evolution [41]. Since NU-RR is somewhat inherently coupled, using the CCD to assess the coupling coordination relationship with NU-RR is scientific. The steps involved in a CCD computation are as follows:

$$C=2\cdot {\left[\frac{U_{1}\cdot U_{2}}{{\left(U_{1}+U_{2}\right)}^{2}}\right]}^{1/2}$$ (6)

$$T=aU_{1}+bU_{2}$$ (7)

$$D=\sqrt{C\times T}$$ (8)

Where *U*_*1*_ is the overall index of new urbanization, *U*_*2*_ is the overall index of rural revitalization; *C* represents the coupling degree; *D* is the coordination degree; *T* is the comprehensive coordination index between systems; The values of a and b are set to equal, both being 0.5, by the recommendation made in the report of the 19th Party Congress to integrate urban and rural development. *D* has a value between 0 and 1. The greater the value of *D*, the better the coupling coordination between NU-RR in the YRB. Referring to relevant research findings [42, 43], the CCD of NU-RR is divided into four stages (Table 3).

**Table 3:** Classification of CCD.

Level	Degree
(0, 0.4]	Low coordination
(0.4, 0.5]	Moderate coordination
(0.5, 0.8]	Highly coordination
(0.8, 1.0)	Extreme coordination

#### 3.2.3 RDD model

Although the CCD model can evaluate the coupling coordination level of NU-RR, it cannot accurately assess the relative development status between the two. So, this paper introduces the RDD model to measure NU-RR’s advanced or lag-related development. The following is the precise formula:

$$\beta =U_{1}/U_{2}$$ (9)

Where: *β* denotes the relative development degree; The complete indices of new urbanization *U*_1_ and rural revitalization *U*_2_.

The reference standards are shown in the Table 4.

**Table 4:** Relative development classification standards.

Level	Degree
0<β≤0.8	New urbanization lag
0.8<β≤1.2	Systematic balanced
1.2<β	Rural revitalization lag

#### 3.2.4 Gravity model

The gravity model is based on Newton’s gravitational equation of gravity, which is primarily applied to studying spatial interactions. It has been involved in many fields, such as transportation, tourism, immigration, and urban analysis [44]. Applying the gravity model to study geographical phenomena allows it to analyze the spatial interaction between cities. This paper attempts to build a coupled and coordinated gravity model from the perspective of spatial linkages, drawing on the primary form of the Newtonian gravitational model, to fully understand the spatial interaction potential of the CCD of NU-RR in the YRB [45]. The equation reads as follows:

$$R_{ij}=k\frac{M_{i}M_{j}}{D_{ij}{}^{2}}$$ (10)

$$R_{i}=\sum_{j=1}^{n}R_{ij}$$ (11)

Where: *R*_*ij*_ denotes the degree of spatial linkage coordination between cities *i* and *j*; *R*_*i*_ stands for the total of the province *i* spatial linkages to the other provinces in the YRB; *M*_*i*_ and *M*_*j*_ denote the coupling coordination values of cities *i* and *j*; *D* for the distance between them; *k*, which is typically 1, for the gravitational constant.

#### 3.2.5 Geographical detector

A statistical method called the geographical detector can reflect the spatial disparity of geographical elements and identify the underlying reasons for those features [46]. This study explores the formation mechanism of the CCD of NU-RR in the YRB using factor probing and interaction probing. The formula is as follows:

$$q=1-\frac{\sum_{h=1}^{L}N_{h}\sigma_{h}{}^{2}}{N\sigma^{2}}$$ (12)

Where: *q* is the detection power value of the detection factor *X*, takes the values [0, 1]; *h* = 1, …*L*, is the stratification of the variable *X*; *N* and *N*_*h*_ are the number of samples within the research area and the detection area; *σ*^2^ and $\sigma_{h}^{2}$ the variance of Y values in the detection area and the study area.

## 4. Analysis of research results

### 4.1 Analysis of NU-RR index system

#### 4.1.1 Comprehensive level analysis of new urbanization

**Fig 3:**
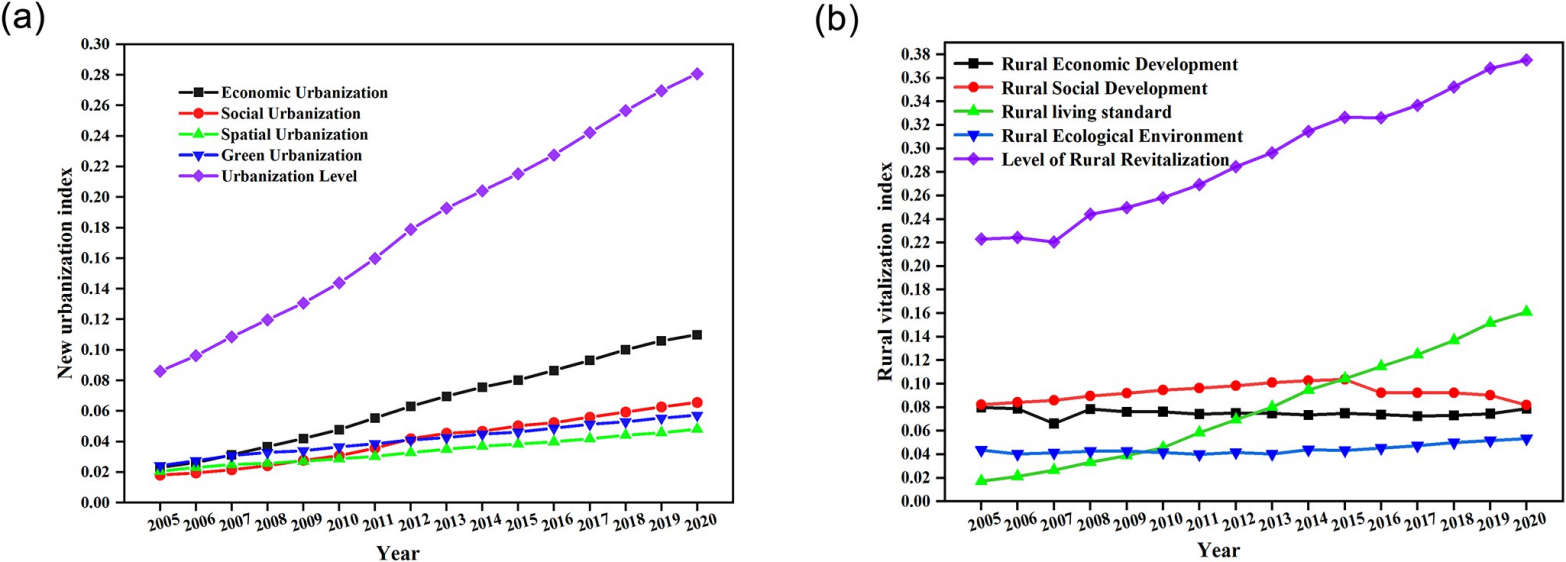
The average level and composite index of new urbanization (a) and rural revitalization (b).

The amount of economic, social, spatial, and green urbanization in the YRB exhibits a rising trend year by year from 2005 to 2020, as shown in Fig 3(a). At a growth rate of 67.9%, the total index of new urbanization increased from 0.09 in 2005 to 0.28 in 2020. This growing tendency is mainly caused by the YRB’s robust economic expansion, which has led to the growth of disposable income per capita and investment in auxiliary infrastructure. The 12th Five-Year Plan’s continued promotion of coordinated regional development and healthy urbanization growth may cause the new urbanization index growth rate’s tiny fluctuations in 2012. Economic urbanization has the highest growth rate of new urbanization indexes, followed by social and green urbanization, while spatial urbanization has the slowest growth rate, indicating that economic urbanization has produced impressive results. However, the construction of green and spatial urbanization still needs to be improved. Among them, social urbanization contributes more, but its growth rate is slower, which indicates that there still needs to be an improvement in infrastructure construction and people’s life security in the YRB. In recent years, the state has actively encouraged the growth of central cities and urban agglomerations in the YRB and has created development plans for each urban agglomeration. These regulations will support the YRB’s new development process even more.

#### 4.1.2 Comprehensive level analysis of rural revitalization

As shown in Fig 3(b), the rural economic development, social development, living standards, and ecological environment in the YRB from 2005 to 2020 all show different change characteristics. The rural vitalization index in the YRB shows a fluctuating rising trend due to the extensive impact of various subsystems. With a growth rate of 42.1%, the total indicator of rural revitalization increased from 0.22 in 2005 to 0.38 in 2020. In October, 2017, General Secretary Xi Jinping pointed out in the report of the 19th Party Congress that the “three rural problems” are fundamental issues related to the country’s livelihood, and that rural development should be given top priority, which has given a boost to the rural construction of the YRB. From the growth trend of rural revitalization indicators, the rural economic development and ecological environment index show a horizontal development trend, which indicates that rural economic and ecological construction is the crucial cruxes affecting rural revitalization in the YRB and have high growth potential. Rural living standards also show an upward trend as rural residents’ per capita net income and living consumption expenditure gradually increase, and the quality of life is improving. Rural social development showed a decreasing trend in 2015, probably due to the accelerated rural labor migration with the accelerated urbanization process, which led to rural labor outflow and hindered rural development.

### 4.2 The coupling and coordination results of NU-RR

#### 4.2.1 Time-series variation of coupling coordination

NU, RR, T, C, and D in the YRB from 2005–2020 were calculated using the above methods. As shown in Table 5, the findings were summarized to determine the mean values for each year.

**Table 5:** CCD from 2005 to 2020.

Years	NU	RR	T	C	D	Coordination category
2005	0.0861	0.2229	0.1545	0.8966	0.3722	Low degree coordination
2006	0.0962	0.2244	0.1603	0.9166	0.3833	Low degree coordination
2007	0.1086	0.2204	0.1645	0.9405	0.3934	Low degree coordination
2008	0.1197	0.2441	0.1819	0.9397	0.4134	Moderate coordination
2009	0.1308	0.2498	0.1903	0.9498	0.4251	Moderate coordination
2010	0.1438	0.2582	0.2010	0.9586	0.4389	Moderate coordination
2011	0.1598	0.2692	0.2145	0.9669	0.4554	Moderate coordination
2012	0.1788	0.2846	0.2317	0.9736	0.4750	Moderate coordination
2013	0.1927	0.2964	0.2446	0.9773	0.4889	Moderate coordination
2014	0.2041	0.3148	0.2595	0.9770	0.5035	Highly coordination
2015	0.2151	0.3266	0.2709	0.9786	0.5149	Highly coordination
2016	0.2275	0.3261	0.2768	0.9840	0.5219	Highly coordination
2017	0.2422	0.3369	0.2896	0.9866	0.5345	Highly coordination
2018	0.2565	0.3524	0.3044	0.9875	0.5483	Highly coordination
2019	0.2697	0.3683	0.3190	0.9880	0.5614	Highly coordination
2020	0.2808	0.3753	0.3280	0.9896	0.5698	Highly coordination

(1) The composite index of NU-RR displays an uptrend. The growth trajectory is always between the development level of NU-RR, showing that the influence of NU-RR on the T value of the YRB is the same. (2) From the coupling degree, the development trends of NU-RR in the YRB are primarily stable and highly coupled during the study period, indicating that cities and villages have strong interaction. (3) From the perspective of CCD, the CCD of NU-RR in the YRB has been improving consistently, and the advancement is positive overall. From 2005 to 2020, the CCD has risen from 0.37 to 0.57, experiencing a development process from “low degree coordination” and “moderate coordination” to “highly coordination”. From 2005 to 2007, they were in a state of poor coordination, which may be related to the fact that the YRB, a crucial region for reducing poverty in China, has yet to properly address poverty issues such as the large number of rural poor and the high proportion of people living in deep poverty, resulting in a lack of effective interaction mechanisms between urban and rural areas. From 2008 to 2013, they were in an intermediate coordination state. From 2014 to 2020, they were highly coordinated, mainly thanks to the implementation of the National New Urbanization Plan (2014–2020), which has boosted urban and rural development.

#### 4.2.2 Spatially divergent features of CCD

**Fig 4:**
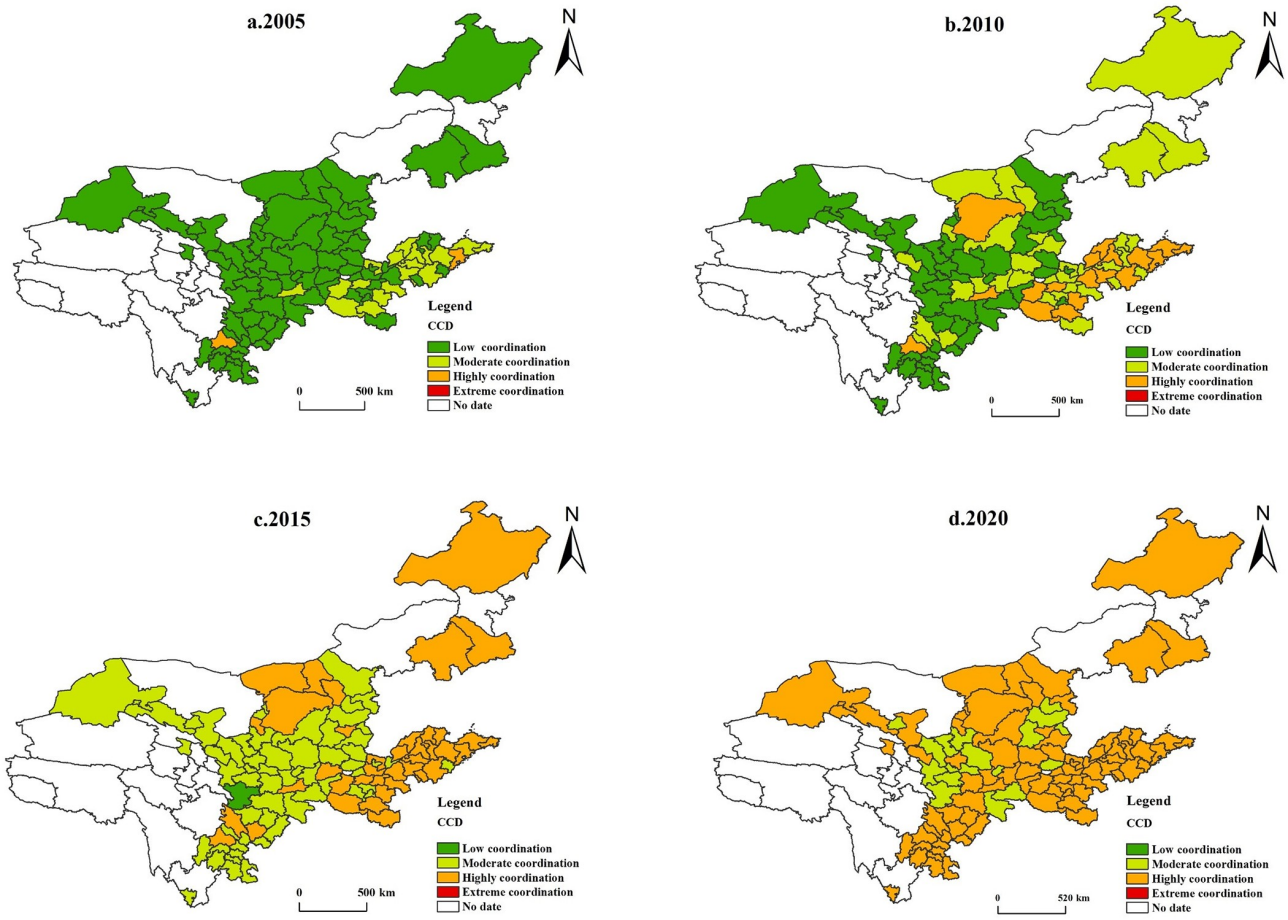
Spatial and temporal pattern of the CCD of NU-RR in the YRB.

To investigate the spatial distribution features of the CCD of NU-RR in the YRB. 2005, 2010, 2015, and 2020 are chosen as the years for analysis. Arc GIS 10.8 software is used to spatially display the data from the four years in the YRB (Fig 4). The following spatial characteristics are displayed:

1. The CCD of NU-RR demonstrates a good development trend, mainly manifested by the increasing number of highly coordinated cities, whose proportion rose from 2.02% in 2005 to 81.8% in 2020, with an increased rate of 79.78%. The number of low and moderately coordinated cities shows a decreasing trend, and the proportion of low-coordinated cities decreases the most, reaching 100%. However, few cities have attained extreme coordination, and there is still space to enhance the YRB’s growth of urban-rural integration.
2. Regional differences in the CCD of NU-RR are weakened, manifesting as medium and highly coordinated urban expansion and low coordinated urban contraction, showing a relatively balanced development trend. From 2005 to 2020, moderate and highly coordinated cities continued to emerge. After Chengdu and Qingdao, highly coordinated cities expanded from downstream cities such as Yantai, Weihai, Jinan, and Zhengzhou to middle and upstream cities, now covering almost the entire YRB. In contrast, the scope of low-coordinated cities has gradually shrunk from the middle and upper reaches to the downstream regions. Currently, there is no low-degree coordinated city, and the whole basin presents a moderate and highly coordinated development trend.
3. By basin, the downstream cities change less, forming a structural feature with Qingdao as the centre and gradually radiating outward. By 2020, all downstream cities will have achieved excellent coordination. The coupling coordination in the midstream cities has changed, evolving from low degree coordination and moderate coordination to high coordination, with only the northern part of Shanxi and part of Gansu in medium coordination in 2020, while the rest of the cities have achieved high coordination. The changing trend of upstream areas is also significant, among which Chengdu has the most developed economy and reached a high level of coordination in 2005, while Longnan city lags and is still at a low level of coordination in 2015, mainly due to its closed geographical environment, natural resource conditions, location, transportation, and economic development level lagging, which seriously hinder the development of urban-rural integration.

#### 4.2.3 Spatially divergent features of RDD

**Fig 5:**
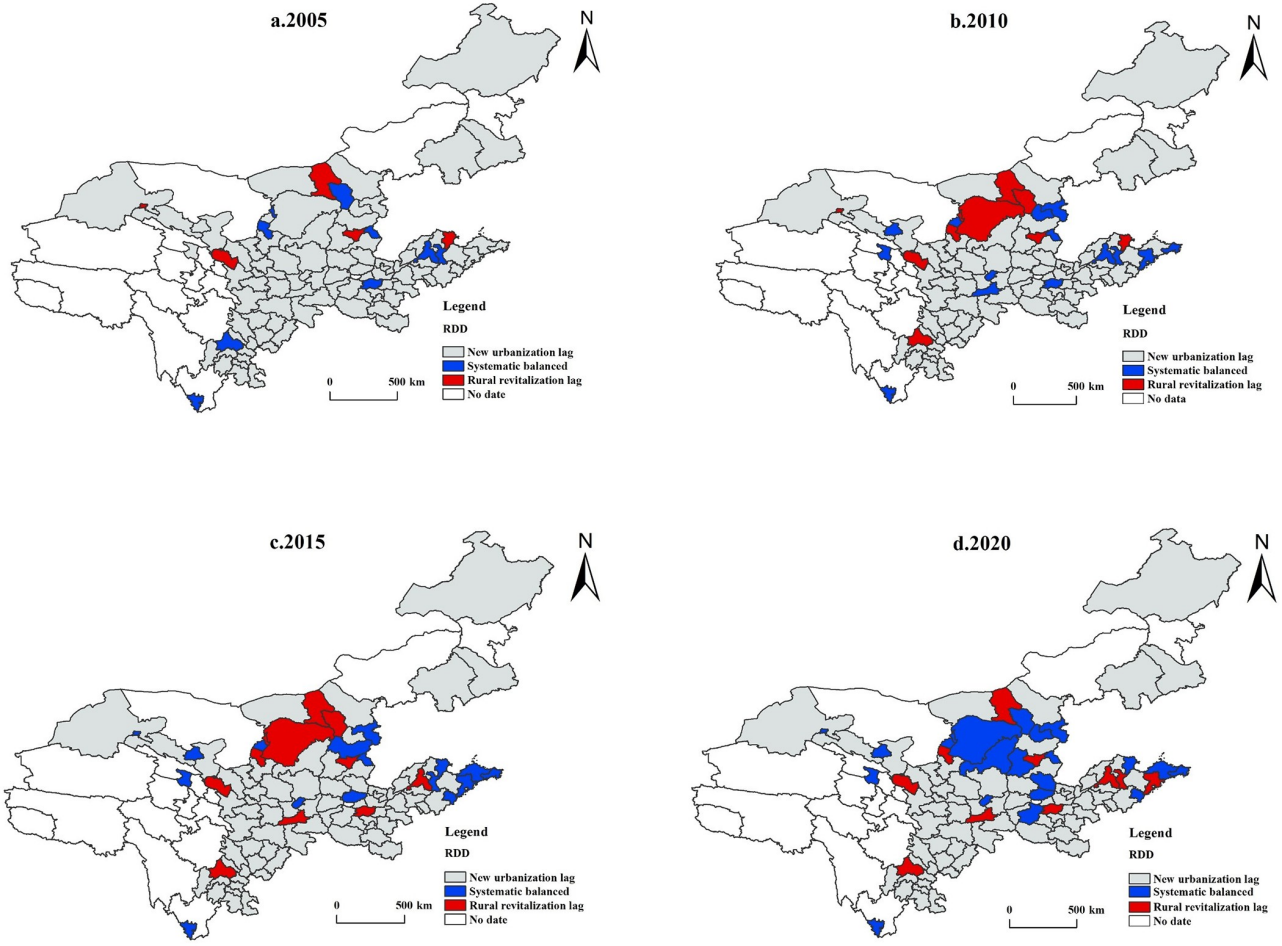
Spatial distribution of the RDD of NU-RR in the YRB.

According to the spatial distribution of the relative development types of NU-RR in the YRB (Fig 5), the spatial evolution characteristics of 2005, 2010, 2015, and 2020 are analyzed. As follows:

1. The RDD of NU-RR is generally in a benign development stage. The primary manifestation is that the number of cities with NU-RR simultaneously developing increases, its proportion grew from 9.09% in 2005 to 21.21% in 2020, and the decrease percentage reached 17.17%. The cities with lagging rural revitalization have no prominent characteristics in terms of quantity.
2. Cities lagging in rural revitalization show a certain degree of regional lock. From 2005 to 2020, the cities lagging in rural revitalization are permanently distributed in Lanzhou, Baotou, Taiyuan, and Chengdu. The rural construction of these cities always needs to catch up to the urban development, indicating that these cities have earlier developed economic conditions and more muscular economic strength. Urbanization will inevitably have a siphoning effect on resources, talents and other factors in the countryside, thus inhibiting the ability of rural areas to develop themselves.
3. The spatial differences in different time dimensions are significant. In 2005, the new urbanization lagging cities occupied almost the whole YRB. Since China’s urbanization development was still in its early stages, urban construction was relatively slow. The abundance of natural resources in rural areas was a growth catalyst for rural development. The spatial characteristics of 2010 and 2015 showed no significant change. Shuozhou, Zhengzhou, and Xi’an have changed from synchronous development to lagging new urbanization, while Xinzhou and Jincheng have changed from lagged rural revitalization to balanced development of both. Most synchronous growth cities in 2020 were in the YRB’s middle reaches. Rural revitalization lagging cities were scattered in various provinces, and the number of new urbanization lagging cities was reduced. However, they still occupy most of the YRB.

#### 4.2.4 Spatial connection of CCD

**Fig 6:**
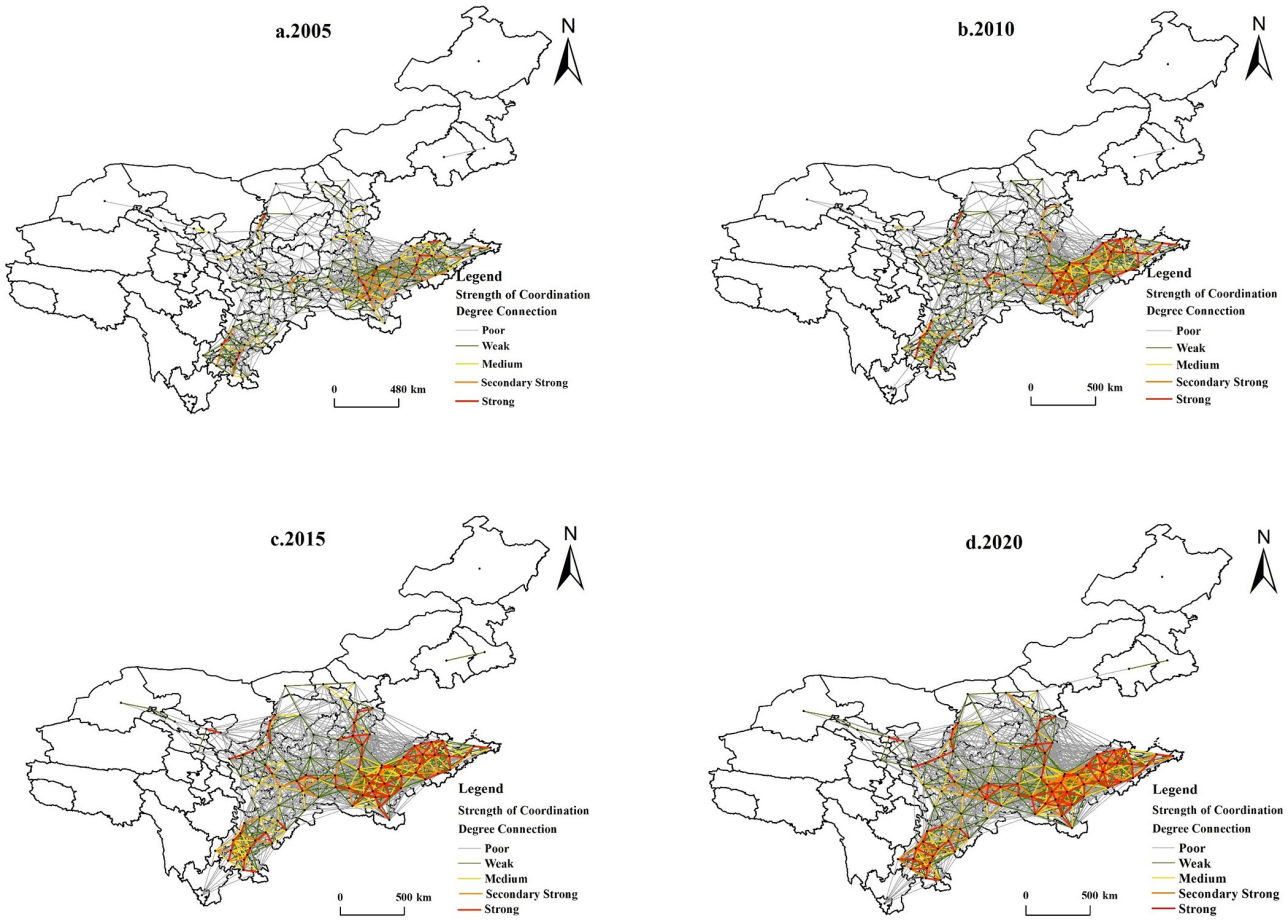
Spatial structure evolution of NU-RR linkages in the YRB.

This study calculates the coupling and coordination link strengths of 99 prefecture-level cities in the YRB for 2005, 2010, 2015, and 2020 using the gravity model to understand better the spatial connectivity network features of the CCD of NU-RR. To better visualize the presentation, the values of gravitational force higher than one are screened to analyze the network connection ability of CCD among cities. From Fig 6, it can be seen that the spatial connection of the CCD of NU-RR in the YRB shows the following characteristics:

1. Evolution from point-axis to group-type spatial structure. The intensity of coupled and coordinated spatial linkages among cities in the YRB gradually increased from 2005 to 2020, and the connection network showed complex structural characteristics. In 2005, 27 groups of cities, such as Yantai-Qingdao, Binzhou-Dongying, and Dezhou-Jinan, were closely connected, while the rest were not. In 2020, the coupling coordination and connection strength among cities will have significantly increased, with more than 100 groups of highly connected cities and a stable triangular structure such as Taiyuan-Yangquan-Jinzhong and a radial structure with developed cities such as Jinan, Zhengzhou, and Chengdu as the core nodes have been formed.
2. The provincial barrier effect gradually weakened. The interaction between cities is steadily enhanced, showing the characteristics of inter-provincial spatial structure. In 2005, the spatial scope of the coupled and coordinated spatial connection between cities radiated the features of adjacent hinterland structures; the spatial tightness effect is significant, but the cross-provincial link is weak. As time evolves, the regional differentiation pattern is easing. By 2020, the number of cities with inter-provincial connections will gradually increase. However, connecting cities in nearby provinces is only possible because of geographical limitations. Cities upstream and downstream of the YRB are not yet sufficiently connected.
3. The spatial polarization effect is significant. From 2005 to 2020, due to the disparity in the economic growth levels of cities in the upper, middle, and lower reaches, the lower reaches’ agglomeration degree of spatial connectivity enhanced, and the spatial agglomeration effect was significant, followed by cities in the middle reaches. In contrast, the downstream towns, except for Sichuan Province, have gradually strengthened their linkages, while cities in the rest of the provinces are relatively weak. The trickle-down effect of the downstream cities should be fully exploited in the future, establish a “one-to-one” city support mechanism and cross-regional cooperation mechanism, and promote the spatial linkage and synergistic development in the east, middle, and west of the YRB.

## 5. Influencing factors of CCD of NU-RR in the YRB

The CCD is chosen as the dependent variable, and 11 independent variables are chosen that may influence the CCD of NU-RR. Article selection 2005, 2010, 2015, and 2020 as time points, Jenks’ natural best breakpoint grading method stratifies the selected independent variables. After converting it from a numerical to a typological quantity, the main factors affecting the CCD of the two in different years and their temporal variation characteristics are explored with the help of a geographical detector.

### 5.1 Analysis of major impact factors

**Fig 7:**
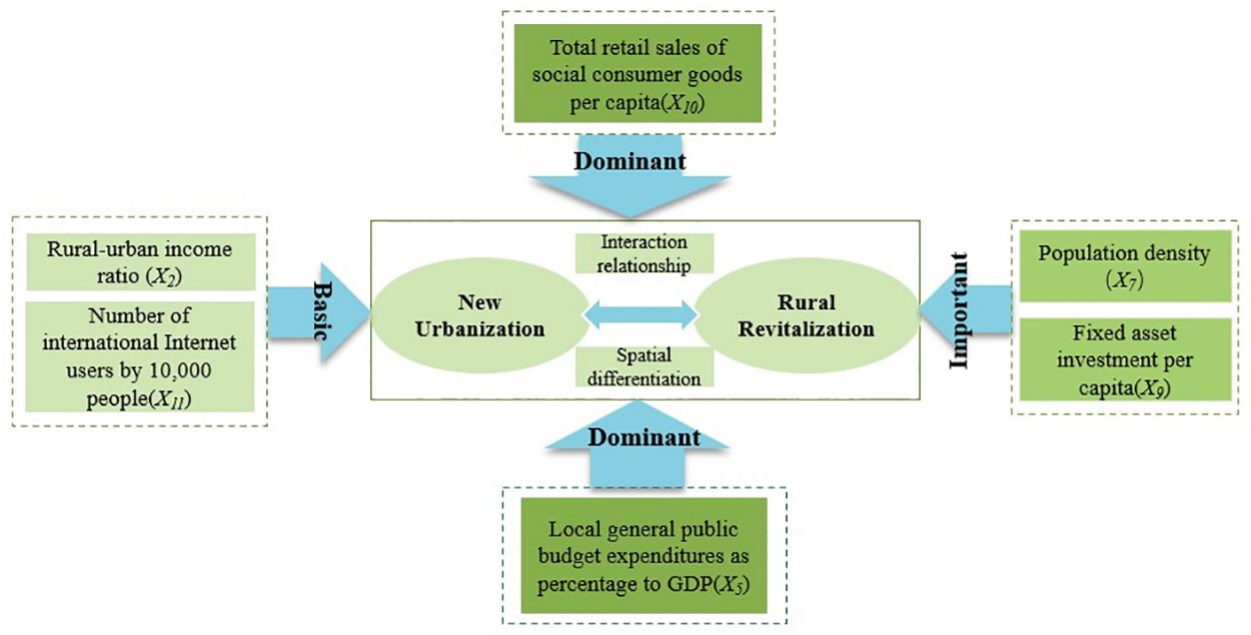
Influence mechanism of NU-RR spatial divergence.

The results of factor detection are shown in Table 6. The factors influencing urban-rural coupling coordination that passed the significance test in 2005 were five factors, ranked according to the magnitude of q values as q(*X*_*10*_), q(*X*_*5*_), q(*X*_*7*_), q(*X*_*9*_), and q(*X*_*11*_). Among them, total retail sales of consumer goods per capita (q>0.4) is the primary influencing factor. The proportion of local general public budget expenditure to GDP, population density, and fixed asset investment per capita (q>0.3) are significant influencing factors. The number of international Internet users has the weakest explanatory power and is an essential influencing factor. After 10 years of development, the changes in 2015 are q(*X*_*10*_), q(*X*_*5*_), q(*X*_*7*_), and q(*X*_*2*_). Among them, in addition to total retail sales of consumer goods per capita, the share of local general public budget expenditure in GDP also rises as a major influence factor, population density remains an important factor, and the urban-rural income ratio becomes an essential influence factor. q(*X*_*10*_), q(*X*_*5*_), q(*X*_*7*_) in 2020. Compared with 2015, the core and significant impact factors are consistent, and the impact of the urban-rural income ratio is insignificant. Overall, the dominant factors affecting the CCD of NU-RR in the YRB are *X*_*10*_ and *X*_*5*_, the important influencing factors are *X*_*7*_ and *X*_*9*_, and the basic influencing factors are *X*_*2*_ and *X*_*11*_, as shown in Fig 7.

**Table 6:** Influence factors detection results.

Factor	2005		2010		2015		2020	
	q	Rank	q	Rank	q	Rank	q	Rank
*X*_*1*_	0.248		0.322		0.319		0.314	
*X*_*2*_	0.189		0.222**	4	0.214*	4	0.170	
*X*_*3*_	0.076		0.125		0.169		0.169	
*X*_*4*_	0.080		0.145		0.163		0.236	
*X*_*5*_	0.387***	2	0.430***	2	0.428***	2	0.435*	2
*X*_*6*_	0.124		0.115		0.125		0.132	
*X*_*7*_	0.339***	3	0.338***	3	0.317***	3	0.347***	3
*X*_*8*_	0.297		0.248		0.276		0.229	
*X*_*9*_	0.324**	4	0.181		0.126		0.096	
*X*_*10*_	0.419***	1	0.436***	1	0.502***	1	0.453**	1
*X*_*11*_	0.275*	5	0.137		0.151		0.071	

Note:

*, **, ***indicate significant at 0.1, 0.05, 0.01 levels.

Specifically, (1) Total retail sales of consumer products per capita (*X*_*10*_) has the most significant impact on the CCD of the two, with q-values reaching above 0.4 in 2005, 2010, 2015, and 2020, with an increasing explanatory role trend. In recent years, the living standard of urban and rural residents in the YRB has improved significantly, and the consumption capacity has increased significantly, accelerating the circulation of resources, capital and other factors between urban and rural areas and strengthening the development potential of the market environment. (2) Local general public budget expenditure as a share of GDP (*X*_*5*_) has a significant driving effect on both, with the coefficient of action showing steady growth from 2005 to 2020 and only showing a slight fluctuation in 2015, probably caught by the fragile nature of the YRB, the lagging economic development and the extensive area of the poor region, the government increases investment in urban and rural infrastructure, thus alleviating the predicament of rural backwardness. (3) Population density (*X*_*7*_) significantly affects urban-rural coupling coordination, with the explanatory effect first weakening and then increasing from 2005 to 2020. As the phenomenon of “hollowing out” of rural areas intensifies, attracting young and strong laborers to return is essential. The YRB should strengthen policy guidance and financial support to encourage elite talents to return to their hometowns to start businesses and promote rural prosperity. (4) The per capita fixed asset investment (*X*_*9*_), the core growth pole of economic growth, reflects the state’s investment in urban and rural areas to improve infrastructure and optimise livelihood projects, etc. In 2005, the economic development of the areas in the YRB was in its initial stage, so it was necessary to increase the investment in new infrastructure through social fixed asset investment to tap the growth potential and promote regional integrated development.

### 5.2 Interaction factor detection analysis

The interactions among the 11 influencing factors were analyzed using the interaction detector, and the top 5 influencing factors in the interaction ranking were selected for analysis, as shown in Table 7. The results showed enhanced synergistic effects among all the influencing factors, which showed two-factor enhancement and non-linear enhancement. Specifically, the top 5 dominant factors ranked in 2005 are *X*_*7*_∩*X*_*10*_, *X*_*7*_∩*X*_*9*_, *X*_*4*_∩*X*_*5*_, *X*_*7*_∩*X*_*5*_, *X*_*7*_∩*X*_*11*_; in 2010, they are *X*_*7*_∩*X*_*10*_, *X*_*6*_∩*X*_*7*_, *X*_*7*_∩*X*_*9*_, *X*_*5*_∩*X*_*10*_, *X*_*4*_∩*X*_*5*_; in 2015, they are *X*_*7*_∩*X*_*10*_, *X*_*5*_∩*X*_*10*_, *X*_*3*_∩*X*_*5*_, *X*_*4*_∩*X*_*5*_, *X*_*2*_∩ *X*_*10*_; in 2020 it changes to *X*_*7*_∩*X*_*10*_, *X*_*7*_∩*X*_*8*_, *X*_*3*_∩*X*_*5*_, *X*_*1*_∩*X*_*7*_, *X*_*7*_∩*X*_*11*_. The interaction of *X*_*7*_ with other factors significantly improves the explanatory degree of the coupled coordination of NU-RR, and it is worth noting that the interaction between *X*_*7*_ and *X*_*10*_ is consistently stronger over the period 2005–2020. In addition to being closely associated with the main influencing factors, urban-rural integration in the YRB is also related to the share of tertiary industry in GDP, the share of secondary industry in GDP and per capita fixed asset investment, and the combined interactions of these factors together have a solid driving effect on urban-rural integration.

**Table 7:** Interaction factor detection results.

2005		2010		2015		2020	
Dominant interaction factor	Interaction type	Dominant interaction factor	Interaction type	Dominant interaction factor	Interaction type	Dominant interaction factor	Interaction type
*X*_*7*_∩*X*_*10*_	Nonlinear enhancement	*X*_*7*_∩*X*_*10*_	Nonlinear enhancement	*X*_*7*_∩*X*_*10*_	Nonlinear enhancement	*X*_*7*_∩*X*_*10*_	Bivariate enhance
*X*_*7*_∩*X*_*9*_	Nonlinear enhancement	*X*_*6*_∩*X*_*7*_	Nonlinear enhancement	*X*_*5*_∩*X*_*10*_	Bivariate enhance	*X*_*7*_∩*X*_*8*_	Nonlinear enhancement
*X*_*4*_∩*X*_*5*_	Nonlinear enhancement	*X*_*7*_∩*X*_*9*_	Nonlinear enhancement	*X*_*3*_∩*X*_*5*_	Nonlinear enhancement	*X*_*3*_∩*X*_*5*_	Nonlinear enhancement
*X*_*7*_∩*X*_*8*_	Nonlinear enhancement	*X*_*5*_∩*X*_*10*_	Bivariate enhance	*X*_*4*_∩*X*_*5*_	Nonlinear enhancement	*X*_*1*_∩*X*_*7*_	Nonlinear enhancement
*X*_*7*_∩*X*_*11*_	Nonlinear enhancement	*X*_*4*_∩*X*_*5*_	Nonlinear enhancement	*X*_*2*_∩*X*_*10*_	Nonlinear enhancement	*X*_*7*_∩*X*_*11*_	Nonlinear enhancement

## 6. Discussion

1. In the YRB, NU-RR has both realized stable growth, and the CCD of the two is on the rise, which is related to the implementation of a series of urban and rural development strategies in China in recent years. In 2020, the CCD of urban and rural areas entered a moderate and highly coordinated state, which is inconsistent with the findings of Wei et al. [47], who showed mainly coordinated and barely coordinated results. This may be due to the different selection of indicators and the different types of coupling coordination. Interestingly, the relative development types of NU-RR in the YRB are dominated by lagging new urbanization, and the deep reasons leading to its formation are worth exploring in our follow-up.
2. Urban-rural integration within a single region is vulnerable to development blockages; only cross-regional mobility can promote sustainable urban and rural development. The gravity model is widely used in the study of inter-urban economic linkages and spatial patterns [48], and it is a helpful attempt to apply it to the study of CCD between two major systems. Ma et al. [45], by constructing a gravitational model of CCD, concluded that the spatial connection strength of CCD of tourism urbanization and ecological environment in 12 western provinces exhibited structural characteristics of complexity and showed a serious bifurcation. This is similar to the results of this paper, probably due to the common features of geographic locations in the study area. Qingdao, Yantai, Zhengzhou, Xi’an, Chengdu, and other cities with comparatively advanced economies and advantageous transportation infrastructure have closer urban-rural spatial connections. Longnan, Tianshui, Guyuan, and other cities are limited by their remote geographical location, weak urban-rural development foundation, and distance from economically developed cities, which lead to weaker links between them.
3. The TOE theoretical framework is mainly applied in urban governance [49] and government public part reform [50], and is less involved in constructing an index system of influencing factors, which helps to provide a new perspective for studying influencing factors. The factors influencing the coupling of NU-RR were discussed using a geographic detector, which, unlike the Tobit model [51] and geographically weighted regression analysis, focused more on detecting stratified heterogeneous space and effectively avoided the interference of spatial complexity. Total retail sales of social consumer goods per capita and the share of local general public budget expenditure in GDP are the core driving factors. It shows that the integrated development of NU-RR is a systematic project, which requires both the decisive role of the market in resource allocation and the promotion of government forces.
4. The above results are based on this article’s comprehensive evaluation index system. The index system construction still needs to be comprehensive because it is more challenging to collect the indicators in rural areas and the missing data in individual years, which affects the analysis results of the data to a certain extent. In the future, it is necessary to enrich and improve the index system and refine the classification stage of CCD to assess the relationship between NU-RR more accurately. In addition, this paper is a study conducted at the municipal level, and future research should be conducted from a more microscopic perspective, at the county scale, to enhance the reliability and relevance of the research results.

Based on the above empirical study discussions, the below countermeasures are put forward to promote high-quality integrated urban and rural development in the YRB:

1. Give full play to the aggregation and radiation capacity of the central cities. Strengthen the leading and radiation-driven role of central cities such as Zhengzhou, Xi’an, Jinan and Qingdao, focus on cultivating the development of provincial capitals such as Xining, Lanzhou, Yinchuan, Hohhot and Taiyuan, strengthen the complementary advantages of central cities and neighboring cities, and form a multi-center, multi-node urban spatial structure; clarify the functional positioning of city groups such as Shandong Peninsula City Cluster, Central Plains City Cluster and Guanzhong Plain City Cluster, optimize city groups functional division of labor, integration of urban and rural advantageous resources, play the overall collaborative effect; large cities to drive large rural areas, promote the rural quality resources and recreation and agricultural tourism depth of integration, to create a number of modern agriculture, tourism, leisure and entertainment, beautiful countryside construction in one sustainable development of the field complex.
2. Strengthen inter-city linkage and cooperation. The YRB should break the administrative boundaries, improve the regional interaction and cooperation mechanism, speed up the interconnection of rail transportation, communication network, environmental protection and other infrastructure construction, enhance population concentration and industrial collaboration, and realize the more extensive scope of spatial linkage and synergistic development; take network integration as a guide, accelerate the construction of “city cluster-metropolitan area-central city-county city” with network integration as a guideline, we will accelerate the construction of a multi-level and networked system of regional high-quality development growth poles, and further promote the connection between cities.
3. The government and the market work in tandem. The government should continuously improve the policy coordination mechanism, deepen the reform of land, finance, household registration and other policy systems, improve infrastructure level, and realize parity of service between urban and rural areas. It should implement policy tilts in the middle and upper reaches of the YRB, accelerate the construction of modern agricultural demonstration zones and industrial parks, create demonstration townships and villages for rural revitalization, improve the rural living environment, and perfect the urban and rural functional systems; deepen market-oriented reforms, implement a unified market access system and standards, and promote the cross-regional flow and optimal allocation of labor, capital, technology and other factors.

## 7. Conclusion

Taking the YRB as the research object, the evaluation index system of NU-RR is comprehensively constructed, and the CCD model, RDD model, gravity model and geographical detector are used to analyze the spatial and temporal characteristics and influencing factors of the CCD of NU-RR in the YRB. The main findings are as follows:

1. From 2005 to 2020, the total index of new urbanization indicates a growing tendency of low starting positions and rising quickly, from 0.09 to 0.28. The overall index of rural revitalization reveals a varying trend of high beginning points and slow progress, ranging from 0.22 to 0.38.
2. The CCD of NU-RR in the YRB continues to rise, showing a spatial pattern of “high in the east and low in the west”. The CCD development stage has evolved through a process of the “low coordination-moderate coordination-high coordination”. The urban-rural coordination in the YRB in 2020 is already moderately and highly coupled, with the CCD of Qingdao and Chengdu being highly coordinated in the long term.
3. The relative development type of NU-RR in the YRB mainly manifests by the lagging new urbanization.
4. The spatial connection strength of CCD exhibits an annual growth pattern, and the degree of connections shows an intricate network structure. The spatial relation of CCD forms a chain radiation network with Shandong, Henan, and Sichuan as the core. In contrast, the other regions, especially Gansu, Ningxia, and Qinghai, do not form a network core with a high radiation effect. However, the interaction between cities is gradually enhanced, and the downstream towns show the characteristics of inter-provincial spatial linkage.
5. The driving factors of the CCD between NU-RR in the YRB are intricate and diverse. The main influencing factors are the total retail sales of consumer goods per capita and local general public budget expenditure as a share of GDP. The influence of any influencing factor after interaction detection is greater than that of a single factor.

## Supporting information

S1 File (ZIP)
